# The King's Fund and Hospital Medical Schools

**Published:** 1910-12-17

**Authors:** 


### The Hospital

A Journal of

The Medical Sciences and Hospital Administration.

New Series. No. 201, Vol. VIII. [No. 1273, Vol. XLIX.] SATURDAY, DECEMBER 17, 1910.

### THE KING'S FUND AND HOSPITAL MEDICAL SCHOOLS.

The correspondence which we have published between the Metropolitan Radical Federation and the honorary secretaries of the King's Fund has excited very general interest throughout the profession and amongst the public. The points raised by the Federation in regard to grants to Metropolitan hospitals with medical schools crystallised down to a charge that the King's Fund, by making a grant to St. George's Hospital in 1909, had condoned the action of St. George's Hospital in "openly flouting the directions of their own distinguished Committee of Inquiry," known as Sir Edward Fry's Commission. The honorary secretaries clearly proved in their reply that this charge was made under a misapprehension, and that in fact there is no ground for any such contention on the part of anybody. It will be observed that in his speech Prince Alexander of Teck, at the meeting of the King's Fund on Wednesday, declared it to be the intention of the Council to uphold the findings of Sir Edward Fry's Commission in this matter, both as to the letter and the spirit. The report of the Distribution Committee confirms the contention of the honorary secretaries of the King's Fund by recommending that the grant to St. George's Hospital this year shall be retained until the King's Fund is satisfied that the financial relations between hospital and school are in accordance with the principles intimated by the Fund in March 1909, and that the general account of St. George's Hospital in respect of 1909, as well as 1910, is secure from any payment on account of medical education. This report having been adopted by the Council of the King's Fund removes any ground for objection which the Metropolitan Radical Federation, and those who sympathise with it, may have thought that they had against the King's Hospital Fund on this point.

The whole question involved by the actual findings of Sir Edward Fry's Commission has been misapprehended in some quarters owing to the provision in Appendix III. of that Commission having been overlooked by many critics. Paragraph 5 of this Appendix states: "It must be remembered that the medical schools, in most cases, undertake many specific laboratory examinations solely for the purpose of helping the physicians and surgeons to form correct diagnoses in the exclusive interests of the patients, and that these would have to be paid for by the hospitals in any case. Where such examinations are made in a laboratory entirely maintained by a school it is obvious that in any exact assessment of the payments and receipts on either side they would have to be taken into account." It is essential that this important point should be fully appreciated by all who take part in any discussion upon this question, for no modern hospital could be efficient which did not enjoy the full advantage for diagnostic purposes of the specific laboratory examinations referred to. One chief ground why the great voluntary hospitals with medical schools in this country are regarded almost universally as the most efficient in the world is that they represent the maximum of development in the scientific and up-to-date treatment of disease, whereby the speedy recovery and the period of life of hospital patients are largely aided.

The many technical and other questions involved in the discussion which has arisen create difficulties not readily understandable by a layman, which makes any public discussion of them so difficult, in fact, as practically to prohibit an accurate and wise decision on the part of anyone not technically familiar with every side of the questions raised. We have always maintained in these columns that the cost of medical education should be absolutely distinct from hospital funds and hospital expenditure which relate to the maintenance of our hospitals and the treatment of the patients whom they contain. London has for years been behind other great centres in regard to medical education owing to the existence of a number of competing medical schools which have handicapped the development of this branch of instruction and kept the Metropolis of the Empire from being, as it should be, the centre and heart of medical study and instruction for students throughout the British Empire. This great evil will never be removed until we secure one great medical school in London with ample funds to provide, that, as a teaching centre and a centre of scientific medicine and surgery at its best, it possesses no rival in the civilised world. The thanks of the whole profession are due to Mr. Henry T. Butlin, the President of the Royal College of Surgeons, for raising this question at the meeting of the King's Fund, for it is undoubtedly the feeling of those who are best entitled to give sober judgment in this matter that in no circumstances is it wise or justifiable that the existing medical schools in London shall be subsidised directly or indirectly by the hospitals to which they are attached. The King's Fund has laboured, and will continue to labour, assiduously in upholding the principles laid down by Sir Edward Fry's Commission, and we hope that Mr. Butlin will be enabled to place himself at the head of a movement to inquire into the whole question of medical education in London, with a view to secure the establishment of one great school. This reform must result in the removal, once and for all, of the many abuses and evils which attend the present unsatisfactory and discredited system of a number of rival schools each attached to a particular hospital, and most of which are languishing for want of funds.

