# Erratum

**Published:** 1910-12-24

**Authors:** 


### ERRATUM.

In the first leading article published in our issue of December 17, first column, line 17, the name of Prince Alexander of Teck is printed in error for that of the Duke of Teck.

